# Filoviruses Use the HOPS Complex and UVRAG To Traffic to Niemann-Pick C1 Compartments during Viral Entry

**DOI:** 10.1128/JVI.01002-20

**Published:** 2020-07-30

**Authors:** Yuxia Bo, Shirley Qiu, Rory P. Mulloy, Marceline Côté

**Affiliations:** a Department of Biochemistry, Microbiology and Immunology, University of Ottawa, Ottawa, Canada; b Ottawa Institute of Systems Biology, University of Ottawa, Ottawa, Canada; c Centre for Infection, Immunity, and Inflammation, University of Ottawa, Ottawa, Canada; University of Kentucky College of Medicine

**Keywords:** Ebola virus, filovirus, vesicular trafficking, virus entry

## Abstract

Ebola virus (EBOV) entry requires internalization into host cells and extensive trafficking through the endolysosomal network in order to reach late endosomal/lysosomal compartments that contain triggering factors for viral membrane fusion. These triggering factors include low-pH-activated cellular cathepsin proteases, which cleave the EBOV glycoprotein (GP), exposing a domain which binds to the filoviral receptor, the cholesterol transporter Niemann-Pick C1 (NPC1). Here, we report that trafficking of EBOV to NPC1 requires expression of the homotypic fusion and protein sorting (HOPS) tethering complex as well as its regulator, UV radiation resistance-associated gene (UVRAG). Using an inducible clustered regularly interspaced short palindromic repeats (CRISPR)/Cas9 system, we demonstrated that depletion of HOPS subunits as well as UVRAG impairs entry by all pathogenic filoviruses. UVRAG depletion resulted in reduced delivery of EBOV virions to NPC1^+^ cellular compartments. Furthermore, we show that deletion of a domain on UVRAG known to be required for interaction with the HOPS complex results in impaired EBOV entry. Taken together, our studies demonstrate that EBOV requires both expression of and coordination between the HOPS complex and UVRAG in order to mediate efficient viral entry.

**IMPORTANCE** Ebola viruses (EBOV) and other filoviruses cause sporadic and unpredictable outbreaks of highly lethal diseases. The lack of FDA-approved therapeutics, particularly ones with panfiloviral specificity, highlights the need for continued research efforts to understand aspects of the viral life cycle that are common to all filoviruses. As such, viral entry is of particular interest, as all filoviruses must reach cellular compartments containing the viral receptor Niemann-Pick C1 to enter cells. Here, we present an inducible CRISPR/Cas9 method to rapidly and efficiently generate knockout cells in order to interrogate the roles of a broad range of host factors in viral entry. Using this approach, we showed that EBOV entry depends on both the homotypic fusion and protein sorting (HOPS) tethering complex in coordination with UV radiation resistance-associated gene (UVRAG). Importantly, we demonstrate that the HOPS complex and UVRAG are required by all pathogenic filoviruses, representing potential targets for panfiloviral therapeutics.

## Abstract (precis)

Ebola viruses (EBOV) and other filoviruses cause sporadic and unpredictable outbreaks of highly lethal diseases. The lack of FDA-approved therapeutics, particularly ones with panfiloviral specificity, highlights the need for continued research efforts to understand aspects of the viral life cycle that are common to all filoviruses. As such, viral entry is of particular interest, as all filoviruses must reach cellular compartments containing the viral receptor Niemann-Pick C1 to enter cells. Here, we present an inducible CRISPR/Cas9 method to rapidly and efficiently generate knockout cells in order to interrogate the roles of a broad range of host factors in viral entry. Using this approach, we showed that EBOV entry depends on both the homotypic fusion and protein sorting (HOPS) tethering complex in coordination with UV radiation resistance-associated gene (UVRAG). Importantly, we demonstrate that the HOPS complex and UVRAG are required by all pathogenic filoviruses, representing potential targets for panfiloviral therapeutics.

## INTRODUCTION

Ebola virus (EBOV) and other members of the family *Filoviridae* are highly pathogenic enveloped RNA viruses and are the causative agents of multiple outbreaks of hemorrhagic fever diseases in humans and nonhuman primates, primarily in central and east Africa (1). Recent outbreaks of Ebola virus disease, including the 2013–2016 outbreak in West Africa that resulted in nearly 29,000 reported cases and over 11,000 deaths, have spurred the development of antibody-based therapeutics and vaccines that are currently under clinical review (2–5).

Filovirus virions harbor a characteristic filamentous morphology and are enclosed by a host cell-derived lipid envelope studded with the trimeric viral glycoprotein (GP). The EBOV GP is classified as a class I viral fusion protein, composed of heterodimers of subunits GP1 and GP2. GP1 contains a receptor binding domain shielded by a glycan cap, while GP2 contains a hydrophobic fusion loop, heptad repeat regions, and a transmembrane domain (6–8). GP-mediated fusion requires triggering factors within the host cell, including cleavage of the GP1 glycan cap by host low-pH-dependent cathepsin proteases, and interaction with the filoviral receptor, the cholesterol transporter Niemann Pick-C1 (NPC1), localized in late endosomes/lysosomes (9–14). Due to the late endosomal localization of these triggering factors, EBOV entry is dependent not only on internalization in host cells but also on trafficking to the entry-conducive intracellular compartments (15–17).

Previous work has shown that EBOV entry requires the activity of cellular trafficking factors, such as the PIKfyve-ArPIKfyve-Sac3 phosphoinositide-regulating complex and the homotypic fusion and protein sorting (HOPS) complex, both of which are involved in maturation and fusion of late endocytic compartments (9, 18–21). Many of these trafficking factors were identified in a loss-of-function haploid (HAP) genetic screen performed by Carette et al., including all members of the HOPS complex (9). Using cells deficient in a component of the HOPS complex, VPS33a, they demonstrated accumulation of vesicular stomatitis virus (VSV) pseudotypes bearing EBOV GP in endosomal compartments, indicating a defect in viral fusion and cytoplasmic escape in these cells. However, whether the class C core vacuole/endosome tethering (CORVET) complex, which shares 4 core subunits (C-Vps core) with the HOPS complex (20), is also required for filoviral entry has yet to be determined. In addition, how the HOPS complex is regulated during viral entry is uncharacterized. Interestingly, studies have demonstrated that UV radiation resistance-associated gene (UVRAG) positively regulates endocytic trafficking by directly binding to members of the HOPS complex (22, 23). Furthermore, Pirooz et al. demonstrated that UVRAG is required for endocytic transport of VSV and influenza A viruses (IAV) through interactions with core components of the HOPS complex and SNAREs (24). Whether filoviruses also require UVRAG for entry remains to be determined.

Using an inducible clustered regularly interspaced short palindromic repeats (CRISPR)/Cas9 system, we demonstrated that members of the C-Vps core and HOPS-specific, but not CORVET-specific, subunits are required for entry mediated by all pathogenic filoviral GPs. Furthermore, we showed that UVRAG expression is required for filovirus entry, with evidence that its ability to bind to the HOPS complex is key to its role in viral entry. Taken together, our studies suggest that filoviruses require coordination of the HOPS complex and UVRAG for delivery to NPC1^+^ compartments and efficient viral entry.

## RESULTS

### An inducible CRISPR/Cas9 system to investigate host trafficking factors required for viral entry.

**FIG 1:**
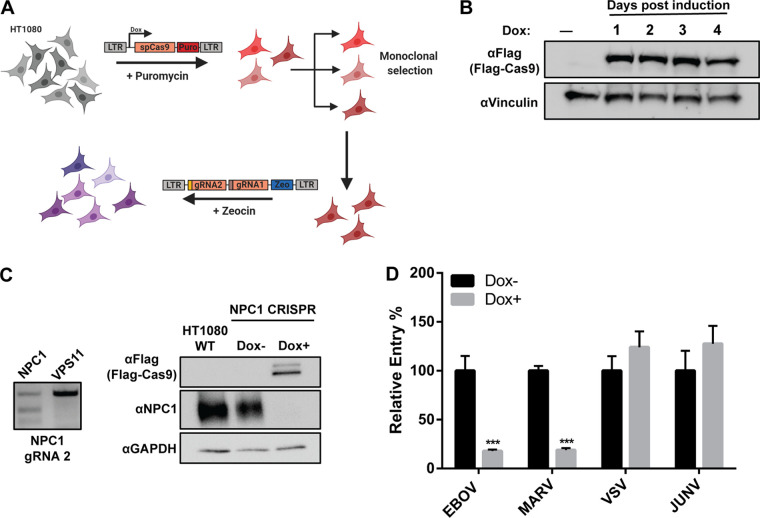
Inducible CRISPR/Cas9 system to investigate host trafficking factors required for viral entry. (A) Schematic of doxycycline (Dox)-inducible CRISPR/Cas9 system using dual gRNAs in HT1080 cells. (B) Time course of Flag-Cas9 expression in CRISPR cells after Dox induction. (C) (Left) Surveyor nuclease assay showing the region of NPC1 targeted by one of the dual gRNAs. (Right) Expression of Flag-Cas9 and NPC1 in NPC1 CRISPR cells after 4 days of Dox induction. (D) NPC1 CRISPR cells were induced for 4 days in Dox, followed by infection with βlam-VLPs harboring EBOV GP, MARV GP, VSV G, or JUNV GPC. Entry was detected by measuring the percentage of cells with cleaved CCF2, normalized to uninduced cells. Results are representative of 3 independent experiments. Asterisks indicate significant differences in entry compared to uninduced cells. ***, *P* < 0.001.

One potential confounding factor with studying vesicular trafficking proteins by generating knockout (KO) cell lines is the development and selection of compensatory mechanisms over several cellular divisions (25). Therefore, to mitigate this problem and investigate the roles of trafficking host factors in viral entry, we designed a CRISPR/Cas9 system by which KOs can be rapidly induced and tested. In this approach, cell lines are engineered to encode Cas9 under the control of a doxycycline (Dox)-inducible promoter and two guide RNAs (gRNAs) targeting the gene of interest (Fig. 1A).

For this study, the human fibrosarcoma cell line HT1080 was chosen, as (i) it is susceptible to filoviral GP-mediated entry and that of other viral glycoproteins, (ii) its flat morphology allows easy visualization and tracking of viral particles by microscopy (18, 26), and (iii) it is pseudodiploid and more karyotypically stable than U2-OS cells, which are also used for microscopy analysis of EBOV trafficking due to their advantageous morphology (15). We first created monoclonal HT1080 cell lines by transduction of a lentiviral vector encoding a Dox-inducible Cas9 and selection with puromycin (27) (Fig. 1A). Following characterization of the clones, one cell line was selected based on its lack of leakiness and high and sustained levels of Cas9 expression following Dox treatment (Fig. 1B). This HT1080 Cas9 monoclonal cell line can then be transduced with lentiviral vectors encoding gRNAs for the gene of interest. To increase the likelihood of KO generation, we generated lentiviral vectors containing two gRNAs for each gene of interest (28) (Fig. 1A). This strategy allows the targeting of different exons, which is valuable when splicing variants are reported and alleviates the impact of one gRNA with low targeting efficiency. The lentiviral vector also carries a zeocin resistance gene, allowing the selection of a polyclonal cell line (Fig. 1A).

As a proof of concept, we targeted NPC1, the filovirus receptor (9, 10, 14), and generated an HT1080 NPC1 CRISPR cell line (Fig. 1A, C, and D). Cas9 expression was induced with Dox, and cells were incubated for 4 days to allow efficient gene editing. To confirm that the gene was edited, we performed a surveyor endonuclease assay. For this, the region of interest was amplified by PCR using primers flanking the gRNA-targeted site and genomic DNA extracted from the induced and noninduced cell populations. The PCR products were then denatured at high temperature and slowly cooled to allow DNA homo- and heteroduplexes to form. Heteroduplexes, which indicate that targeting of the gene resulted in indels, were then digested with T7 endonuclease I. Analysis of the NPC1 genomic DNA regions around the gRNAs using the endonuclease assay revealed that the region was modified for the HT1080 NPC1 CRISPR cells (presence of a cleaved band in addition to the uncleaved band) but not for another CRISPR cell line (Fig. 1C, left; results for gRNA 2 are shown). While this method indicates that the targeting was successful, it does not allow quantitative assessment of knockdown. Therefore, we measured NPC1 expression by immunoblotting and found it to be undetectable following 4 days of Dox stimulation (Fig. 1C, right).

Finally, to assess the potential of this strategy to investigate viral entry host factors, we used virus-like particles (VLPs), which can be generated by the coexpression of the EBOV nucleoprotein and matrix protein (VP40) (29). These VLPs have the characteristic filamentous morphology of filoviruses and can enter cells when viral GPs are coexpressed during production. In addition, using a VP40 construct fused with beta-lactamase (βlam), viral entry and delivery of VP40 into the target cell cytoplasm can be measured after the target cells have been loaded with the βlam fluorescence resonance energy transfer (FRET) substrate CCF2. Dox-induced or noninduced NPC1 CRISPR cells were incubated with VLPs harboring EBOV or Marburg virus (MARV) GPs, or the rhabdovirus vesicular stomatitis virus (VSV) glycoprotein (G), or the arenavirus Junin virus (JUNV) glycoprotein precursor (GPC) as controls. As expected, we found that entry was abrogated specifically for EBOV and MARV VLPs upon NPC1 targeting, while entry mediated by VSV G or JUNV GPC was not affected (Fig. 1D). Taken together, these experiments validate our CRISPR/Cas9 strategy for the rapid and efficient generation of KO cell lines to test the importance and roles of host factors in viral entry.

### HOPS, but not CORVET, is required for efficient EBOV GP-mediated entry.

**FIG 2:**
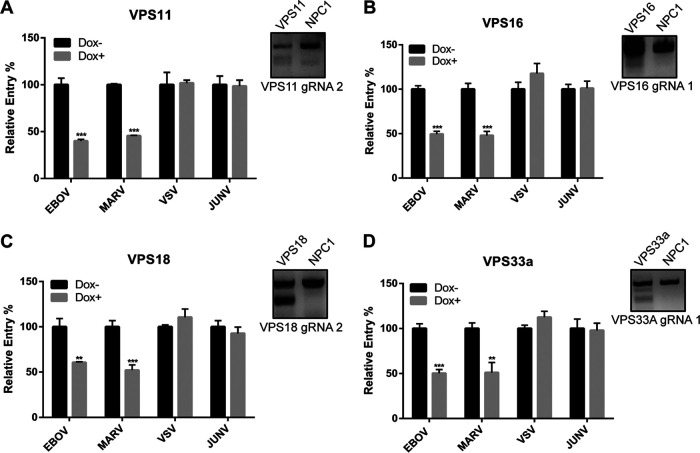
The C-Vps core is required for filovirus entry. VPS11 (A), VPS16 (B), VPS18 (C), and VPS33a (D) CRISPR cells were infected by a panel of βlam-VLPs harboring EBOV GP, MARV GP, VSV G, or JUNV GPC following 4 days of Dox induction. Entry was detected by measuring the percentage of cells with cleaved CCF2, normalized to uninduced cells. Results are representative of 3 independent experiments. Asterisks indicate significant differences in entry compared to uninduced cells. Surveyor nuclease assay results for one gRNA of each targeted gene are shown on the right. **, *P* < 0.01; ***, *P* < 0.001.

**FIG 3:**
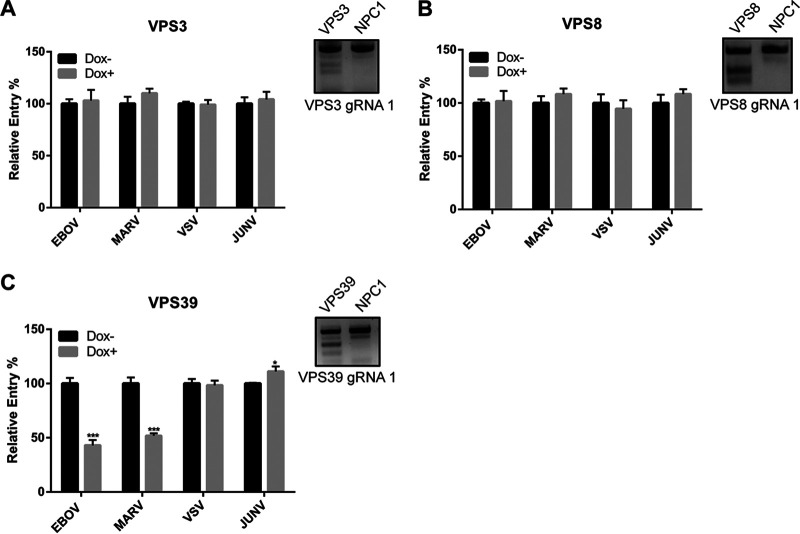
A HOPS-specific, but not CORVET-specific, subunit is required for filovirus entry. VPS3 (A), VPS8 (B), and VPS39 (C) CRISPR cells were infected by a panel of βlam-VLPs harboring EBOV GP, MARV GP, VSV G, or JUNV GPC following 4 days of Dox induction. Entry was detected by measuring the percentage of cells with cleaved CCF2, normalized to uninduced cells. Results are representative of 3 independent experiments. Asterisks indicate significant differences in entry compared to uninduced cells. Surveyor nuclease results for one gRNA of each targeted gene are shown on the right. *, *P* < 0.05; ***, *P* < 0.001.

To further test our system, we first sought to investigate the HOPS complex, as it was previously identified in a screen for EBOV host factors using haploid cells (9). The HOPS complex shares 4 subunits (C-Vps core, composed of Vps11, Vps16, Vps18, and Vps33a) with the CORVET complex (20). In addition, each contains 2 specific subunits (HOPS contains Vps39 and Vps41; CORVET contains Vps3 and Vps8). In order to investigate the importance of the C-Vps core as well the HOPS- and CORVET-specific subunits in filovirus entry, we generated HT1080 CRISPR cell lines targeting the C-Vps core (Fig. 2), as well as Vps39, as a representative of the HOPS complex, and Vps3 and Vps8, the two accessory subunits of the CORVET complex (Fig. 3). Following 4 days of Dox induction of Cas9 expression, genomic DNA targeting was assessed by endonuclease assays (Fig. 2 and 3, insets), and viral entry was assessed using VLPs harboring the glycoproteins of EBOV, MARV, VSV, and JUNV. We found that the genetic targeting of all of the C-Vps subunits reduced viral entry mediated by both EBOV and MARV GP but had no effect on that by VSV G or JUNV GPC (Fig. 2). These results indicate that efficient filovirus entry into cells requires an intact C-Vps core and suggest dependence on the CORVET and/or HOPS complex(es) for entry (Fig. 2A to D).

Therefore, we next investigated subunits exclusive to CORVET (Vps3 and Vps8) or HOPS (Vps39) (Fig. 3A to C). Again, targeting of the genes was confirmed by endonuclease assays (Fig. 3, insets). Using VLPs, we found that targeting the CORVET-specific subunits, Vps3 and Vps8, had no effect on viral entry by any of the EBOV- or MARV-GP VLPs (Fig. 3A and B). In contrast, targeting of the HOPS-specific subunit, Vps39, specifically reduced EBOV and MARV GP-mediated entry (Fig. 3C). Collectively, these data suggest that viral entry in the CRISPR cells targeting C-Vps and a HOPS-specific subunit was consistently reduced for VLPs harboring the GPs of EBOV or MARV, suggesting that the HOPS complex plays an important role in filoviral entry, while the CORVET complex is dispensable. In addition, these experiments indicate that our inducible CRISPR/Cas9 system can be broadly used to study the importance of host trafficking factors in viral entry.

### UVRAG is required for EBOV GP-mediated entry.

**FIG 4:**
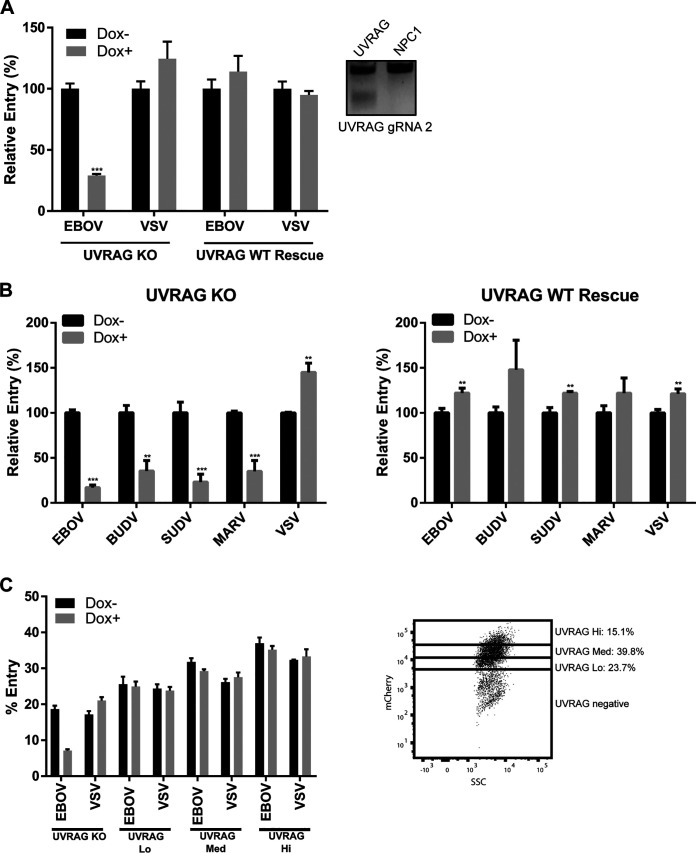
UVRAG expression is required for filovirus entry. (A) UVRAG KO and WT-add-back CRISPR cells were induced in Dox for 4 days, followed by infection with βlam-VLPs harboring EBOV GP or VSV G. Entry was detected by measuring the percentage of cells with cleaved CCF2, normalized to uninduced cells. Results are representative of 3 independent experiments. Asterisks indicate significant differences in entry compared to uninduced cells. Surveyor nuclease results for UVRAG gRNA2 are shown on the right. (B) Infection of UVRAG KO or WT-add-back CRISPR cells by a panel of VLPs bearing different filoviral glycoproteins or VSV G following 4 days of Dox induction. Asterisks indicate significant differences in entry compared to uninduced cells. Results are representative of 3 independent experiments. (C) UVRAG KO and WT-add-back CRISPR cells were induced and infected with VLPs as for panel A. Entry in the low (lo), medium (med), and high (hi) mCherry-UVRAG-expressing cells was determined by mCherry expression (dot plot [inset]). Percent entry was significantly increased for both EBOV and VSV with increasing mCherry expression (one-way analysis of variance [ANOVA], Tukey’s test; *P* < 0.05). **, *P* < 0.01; ***, *P* < 0.001.

Previous work by Liang et al. has shown that UVRAG positively regulates the C-Vps core complex during autophagosome and endosomal maturation and can pull down the HOPS-specific subunit Vps39, indicating that UVRAG plays a role in HOPS activity (22). To investigate whether UVRAG is also required for EBOV GP-mediated entry, we used our inducible CRISPR/Cas9 system and engineered HT1080 UVRAG CRISPR cells (Fig. 4A). Targeting of the UVRAG gene was confirmed by an endonuclease assay (Fig. 4A, inset). Interestingly, we noticed that UVRAG depletion resulted in decreased cell growth (data not shown).

Using VLPs harboring EBOV GP or VSV G, we tested the effect of UVRAG depletion on viral entry. We found that Dox stimulation of the UVRAG CRISPR cells reduced EBOV GP-mediated entry but had no effect on entry of VLPs harboring VSV G (Fig. 4A). To confirm that the decrease in viral entry was specifically due to UVRAG depletion, we engineered the UVRAG CRISPR cells to stably express UVRAG cDNA with silent mutations in the protospacer-adjacent motif (PAM) sequences of both gRNA-targeted sequences. Because the PAM sequences are required for Cas9 binding (30), this UVRAG cDNA is not targeted and can be expressed following Dox treatment. As expected, entry mediated by both EBOV GP and VSV G was unaffected by Dox treatment in the rescued UVRAG CRISPR cells (Fig. 4A), indicating that the specific reduction in entry for EBOV VLPs was due to UVRAG depletion. To test whether UVRAG is required for entry by multiple filoviruses, we generated VLPs harboring the GPs of other pathogenic EBOV species, Bundibugyo ebolavirus (BUDV) and Sudan ebolavirus (SUDV), and MARV. Using those VLPs, we found that entry by all filoviruses tested was decreased in the Dox-induced UVRAG CRISPR cells yet was rescued when UVRAG cDNA was added back (Fig. 4B). These results indicate that UVRAG is required for a filovirus-specific entry step.

Furthermore, the WT UVRAG construct was fused to mCherry, enabling us to examine the effect of the UVRAG expression level on viral entry by gating cells based on mCherry expression. The UVRAG-mCherry positive population was separated into three groups: the low, medium, and high expressors (Fig. 4C). Viral entry in each of these groups was then determined. We found an increase in the percentage of cells with cleaved CCF2 with increasing expression of UVRAG (Fig. 4C), indicating enhanced viral entry when UVRAG was overexpressed. This increase was not specific to EBOV GP-mediated entry, as it was also observed for VLPs harboring VSV G (Fig. 4C). Taken together, these results suggest that UVRAG is a nonspecific positive regulator of viral entry yet is specifically required for filovirus entry.

### EBOV trafficking to NPC1^+^ compartments requires UVRAG.

**FIG 5:**
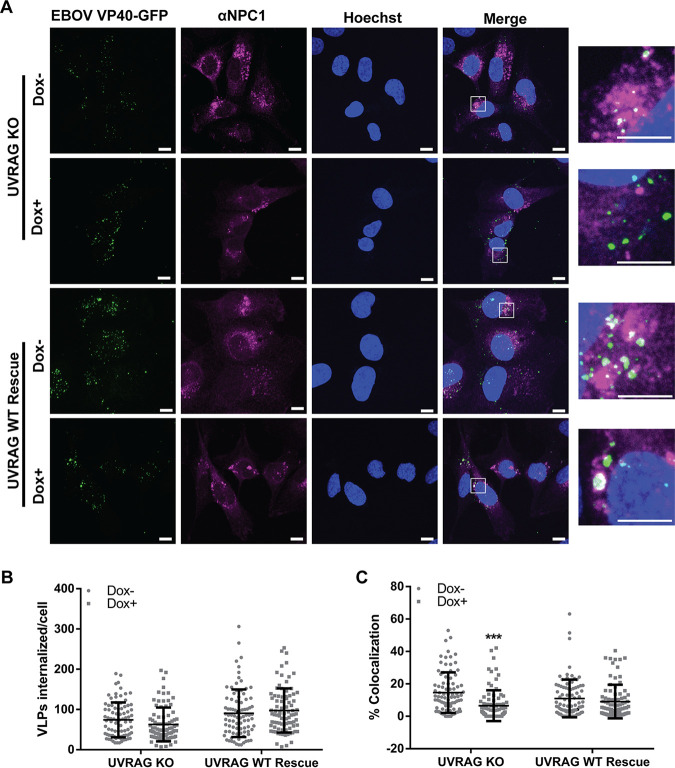
EBOV trafficking to NPC1^+^ compartments is impaired in UVRAG-deficient cells. (A) UVRAG CRISPR KO and WT-add-back cells were induced in Dox for 4 days, followed by reseeding onto coverslips. Cells were then infected with GFP-VLPs harboring fusion-deficient EBOV ΔM GP^F535R^ for 3 h. Cells were fixed, permeabilized, and stained with an NPC1 antibody and Hoechst. Cells were imaged on an LSM800 confocal microscope (Zeiss). The number of internalized VLPs (B) and colocalization between VLPs and NPC1 (C) were analyzed using Imaris software (Bitplane). Results shown are combined normalized data from 3 experiments. Asterisks indicate significant differences in colocalization compared to uninduced cells, as determined by an unpaired *t* test with Welch’s correction, and statistical significance determined using the Holm-Šídák method. ***, *P* < 0.001.

UVRAG is involved in multiple cellular processes, such as autophagosome formation and maturation and endosomal maturation (22, 31). Because all filoviruses require trafficking to compartments containing NPC1 for entry, a simple hypothesis is that UVRAG depletion leads to accumulation of viral particles in early compartments lacking NPC1 due to a failure in endosome maturation. To test this hypothesis, we produced fluorescent EBOV VLPs using VP40 fused to green fluorescent protein (GFP) and fusion-defective EBOV GP^F535R^ to allow viral accumulation in compartments containing NPC1 (18, 32). Following 4 days of Dox induction, UVRAG CRISPR cells were incubated with fluorescent EBOV VLPs at 4°C to allow virus binding to the cell surface, before switching to 37°C to allow internalization and trafficking of viral particles in cells. After 3 h, cells were fixed, stained for NPC1, and visualized by confocal microscopy (Fig. 5A). While we did not observe differences in the numbers of internalized EBOV VLPs in the UVRAG CRISPR cells following Dox stimulation (Fig. 5A and B), we found that colocalization with NPC1 was drastically decreased (Fig. 5A and C). Interestingly, we noticed a slight increase in internalized VLPs in the rescued UVRAG cells, irrespective of Dox treatment (Fig. 5B). Importantly, in the rescued cells, colocalization of VLPs with NPC1 remained unchanged upon Dox stimulation (Fig. 5A and C). Taken together, these data suggest that UVRAG is required for EBOV trafficking to NPC1.

### Deletion of UVRAG domains required for HOPS association impairs EBOV entry.

**FIG 6:**
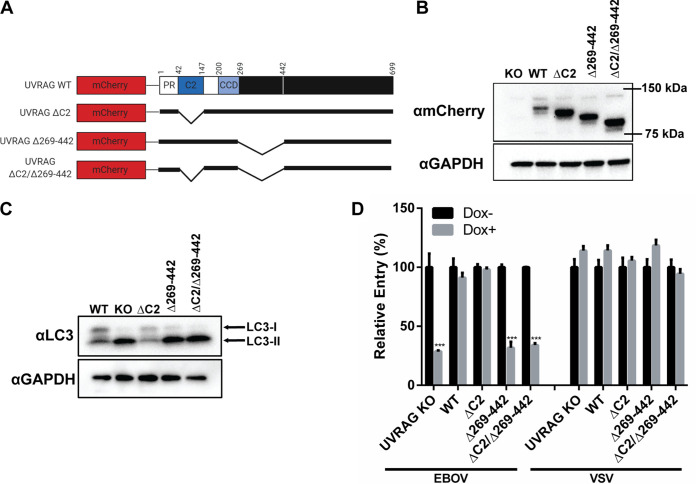
Deletion of UVRAG domains required for HOPS association impairs EBOV entry. (A) Schematic of mCherry-tagged UVRAG deletion constructs. (B) Immunoblot of mCherry-UVRAG in UVRAG CRISPR cells transduced with the deletion constructs. (C) Expression of LC3 in UVRAG CRISPR cells transduced with the deletion constructs was detected by immunoblotting following 4 days of induction in Dox. (D) UVRAG CRISPR KO, WT-add-back, or deletion construct-add-back cells were induced in Dox for 4 days, followed by infection with βlam-VLPs harboring EBOV GP or VSV G. Entry was detected by measuring the percentage of cells with cleaved CCF2, normalized to uninduced cells. Asterisks indicate significant differences in entry compared to uninduced cells. Results are representative of 3 independent experiments. ***, *P* < 0.001.

Our results, obtained by using an inducible CRISPR strategy, suggest that the C-Vps core and the HOPS complex, but not the CORVET complex, are required for filovirus entry (Fig. 2 and 3). In addition, using the same strategy, we showed that UVRAG is required for filovirus entry (Fig. 4), more specifically, for delivery of viral particles to NPC1 compartments (Fig. 5). Because previous studies have shown that endosome fusion and autophagosome-lysosome fusion are mediated by a C-Vps–UVRAG complex (22), it is likely that the function of these proteins is also linked during filovirus GP-mediated entry. Two regions of UVRAG have been shown to interact with the C-Vps core subunit Vps16: the Ca^2+^-dependent phospholipid-binding C2 domain and a region that encompasses amino acids 269 to 442, located in a domain of unknown function (22). To investigate whether expression of these regions is required for UVRAG function and viral entry, we deleted the C2 domain (ΔC2), the middle region (Δ269–442), or both regions (ΔC2/Δ269–442) in the UVRAG PAM-mutated cDNA (Fig. 6A). Using lentiviral vectors, these constructs were transduced in the UVRAG CRISPR cells. All deletion mutants were expressed at similar levels, suggesting that the deletions had no effect on protein stability (Fig. 6B).

UVRAG interacts with C-Vps to perform at least two functions: endosome and autophagosome maturation (22). This process involves the fusion of autophagosomes with late endosomes/lysosomes for degradation of autophagosome contents. A block in autophagosome maturation can be detected by the accumulation of the lipidated form of LC3 (LC3-II), which would otherwise be degraded in the lysosomes (33). To characterize the effect of the UVRAG deletions mutants on this C-Vps-dependent function, we treated the UVRAG CRISPR cells with Dox to deplete endogenous UVRAG and tested the ability of wild-type (WT) UVRAG and the ΔC2, Δ269–442, and ΔC2/Δ269–442 constructs to rescue autophagosome maturation by blotting for LC3. As expected, an increase in LC3-II was observed in the UVRAG-depleted cells, which was rescued when WT UVRAG was expressed (Fig. 6C). Similarly, expression of UVRAG ΔC2 was also able to rescue autophagosome maturation, suggesting that the C2 domain is not required for UVRAG-mediated autophagosome maturation (Fig. 6C). Interestingly, the UVRAG Δ269–442 and ΔC2/Δ269–442 deletion mutants were both unable to rescue autophagosome maturation (Fig. 6C). These results suggest that the 269–442 region is required for UVRAG’s ability to mediate autophagosome maturation in HT1080 cells, while C2 is dispensable for this function.

Next, we sought to investigate whether the UVRAG deletion mutants could rescue EBOV GP-mediated entry. The UVRAG CRISPR cells were treated with Dox and exposed to VLPs harboring EBOV GP or VSV G. As previously shown, the expression of UVRAG WT rescued the inhibition in EBOV VLP entry, while entry mediated by VSV G remained unaffected (Fig. 6D). Similar to its ability to restore autophagosome maturation (Fig. 6C), we found that UVRAG ΔC2 was also able to compensate for endogenous UVRAG depletion in the context of EBOV entry (Fig. 6D). In contrast, UVRAG Δ269–442 and the double-deletion mutant, which could not restore autophagosome maturation (Fig. 6C), did not rescue EBOV VLP entry (Fig. 6D). These data suggest that there are functional similarities in the interactions between UVRAG and C-Vps to perform autophagosome or endosomal maturation. Taken together, these results provide further evidence suggesting that UVRAG is required for EBOV GP-mediated entry.

## DISCUSSION

To infect cells, filoviruses are internalized via macropinocytosis and trafficked to endosomal/lysosomal compartments containing triggering factors required for viral membrane fusion and subsequent delivery of the viral genome into the host cell cytoplasm. In this study, we present an inducible dual-gRNA CRISPR/Cas9 strategy to investigate the roles of host factors in viral entry. We demonstrate that filoviruses require the HOPS, but not the CORVET, complex for entry. In addition, we show that UVRAG is required for filovirus entry, more specifically, for delivery of EBOV particles to intracellular vesicles containing the entry receptor, NPC1. Finally, the ability of UVRAG to mediate EBOV GP-mediated entry depends on a domain known for interacting with the HOPS complex, suggesting that its role in viral entry involves coordination with the HOPS complex. Our findings strengthen the notion that a specific trafficking pathway is required for efficient filovirus entry, more specifically, one that involves the HOPS complex and UVRAG.

Multiple genetic strategies have been used to identify host factors required for viral entry, including RNA interference (RNAi) screens and, more recently, screens based on mutagenized haploid cells and CRISPR/Cas9 (9, 34–36). One major advantage of these methods is the nonbiased discovery of novel genes. However, because of the requirement for multiple days of selection and expansion of initial cell populations, genes required for cell proliferation and survival are generally negatively selected, preventing investigation of their potential roles in viral entry. Here, we used an inducible Cas9 system combined with a dual-gRNA strategy for the specific investigation of the roles of proteins involved in endosomal/lysosomal cargo trafficking. The rapid testing of viral entry after inducing expression of Cas9 and knocking out specific genes makes the study of essential cellular genes possible. For instance, this system allowed us to study the role of UVRAG in filovirus entry. UVRAG’s role in autophagy is very well characterized, yet autophagy-independent functions have also been described (22, 31). While its role in vesicular trafficking was the focus of this study, UVRAG is also involved in cell proliferation and homeostasis (31, 37). The negative effect of UVRAG knockout on cellular proliferation could explain why UVRAG was not identified in the HAP screen for EBOV host factors (9). While the dual-guide strategy does not lend itself to nonbiased screens, using the inducible Cas9 with a single gRNA could allow such screens.

Previous studies have shown that, following internalization, EBOV particles traffic from a Rab5^+^ early compartment to a Rab7^+^ late compartment (38). The passage through a Rab5^+^ vesicle could imply a role for the CORVET complex, a Rab5 effector which has been implicated in early endosome fusion (39). In addition, transition to late compartments implies the use of the HOPS complex. Using the inducible CRISPR/Cas9 strategy, we targeted all members of the C-Vps core which have been found to be localized to both early and late endosomal compartments (20). Interestingly, we found that targeting the subunits of the C-Vps core and those of the HOPS complex specifically reduced entry by the GPs of EBOV and MARV but had no effect on that by VSV G and JUNV GPC, which is in agreement with results reported by Carette et al. (9). In contrast, targeting Vps3 or Vps8 did not affect entry by any of the VLPs, suggesting that filovirus entry does not depend on the CORVET complex (Fig. 3A and B). Previous studies have shown that while the depletion of CORVET-specific subunits has no effect on late endosomal compartments, it alters early-endosomal subpopulations differentially (40). These endosomal populations are characterized by the presence of Rab5 with either early endosome antigen 1 (EEA1) or the adaptor protein phosphotyrosine interacting with PH domain and leucine zipper 1 (APPL1), or both (41). Interestingly, Vps3 depletion seemed to mostly affect APPL1 positive endosomal population, causing its fragmentation (40). While more work is needed to characterize our CORVET CRISPR cell lines, it is possible that if filoviral VLPs, or even those harboring VSV G and JUNV GPC, transit through early endosomal compartments, those early compartments are among the subpopulations that are not coordinated by CORVET. Another possibility is that Vps3 or Vps8 targeting only slows viral trafficking to entry-conducive compartments, and kinetic experiments may be necessary to reveal a role for the CORVET complex in filoviral entry.

Recent studies have shown that UVRAG can coordinate C-Vps and HOPS functions (22). Here, we show that depletion of UVRAG specifically leads to reduced filovirus GP-mediated entry (Fig. 5 and 6). In a previous study, Pirooz et al. showed an increase in VSV and IAV entry when UVRAG was overexpressed and inhibition when UVRAG, Vps16, or Vps18 was depleted (24). While we also observed increased entry upon UVRAG overexpression (Fig. 4C), our results suggest that UVRAG and the C-Vps core are not required for VSV G-mediated entry (Fig. 2 and 4). The C-Vps and HOPS complexes were also shown by Carette et al. (9) to be dispensable for VSV infection. Since VSV G-mediated membrane fusion in the endosome is triggered by low pH, it is possible that the need for VSV to traffic deeper in the endosomal pathway, which is mediated by the HOPS complex and UVRAG, depends on the pH of early endosomes, which is likely cell type specific and can also be modulated by cell-extrinsic factors, such as extracellular pH (42). This could explain differences in the observed dependence on UVRAG and endosomal maturation for VSV entry. In addition to a role in endosome/lysosome fusion, UVRAG is known to be important for both the initiation and maturation of autophagosomes (22, 31). Initiation of autophagosomes requires UVRAG interaction with Beclin, and maturation is dependent on interaction with the C-Vps complex. While a defect in autophagy initiation was not observed in our HT1080 UVRAG CRISPR cells, autophagosome fusion with lysosomes seemed to be impaired, as an accumulation of LC3-II was apparent in the Dox-treated cells (Fig. 6C). The absence of an effect on autophagy initiation in UVRAG KO cells was also reported for lymphocytes (37), suggesting that the role of UVRAG in autophagosome formation may be cell type specific. A recent study reported a role for autophagy-associated proteins, such as Beclin and LC3, in EBOV internalization (43). While we did not observe a reduction in VLP internalization in UVRAG-depleted cells, more work needs to be done to assess the autophagy-dependent functions of UVRAG in EBOV entry. Interaction of UVRAG with C-Vps and the HOPS complex was previously shown to map to the C2 (42–147) and 269–442 regions of UVRAG (22, 24). Interestingly, we found that UVRAG function in viral entry requires the domain comprising amino acids 269 to 442, while the UVRAG with C2 deleted was capable of both infection rescue and autophagosome clearance (Fig. 6C and D), suggesting that this region is not involved in C-Vps and HOPS complex function in HT1080 cells. Instead, in our system, the 269–442 region was critical for UVRAG-mediated autophagosome maturation and virus trafficking (Fig. 6C and D). More work is required to analyze the contribution of this region for UVRAG functions in autophagosome/endosome maturation and viral entry.

In conclusion, our study describes a dual gRNA and inducible CRISPR/Cas9 system to study host factors involved in viral entry. The data we obtained by using this strategy support a model in which filovirus entry requires a specific set of host trafficking proteins for virus delivery to the intracellular entry receptor, NPC1. Better molecular characterization of this conserved trafficking pathway and the identification of additional host proteins involved in its regulation could pave the way to the development of panfiloviral therapeutic strategies.

## MATERIALS AND METHODS

### Cell lines and antibodies.

HEK293T and HT1080 cells (ATCC) were cultured in Dulbecco’s modified Eagle medium (DMEM; Wisent) supplemented with 10% fetal bovine serum (Sigma), 1 U/ml penicillin, 1 μg/ml streptomycin, and 3 μg/ml glutamine (Corning) at 37°C and 5% CO_2_. Antibodies against NPC1 (Abcam), Flag (Sigma), mCherry (Abcam), LC3 (Novus Biologicals), GAPDH (Abcam), and vinculin (Abcam) were used.

### CRISPR cloning and cell lines.

HT1080 cells expressing doxycycline (Dox)-inducible Cas9 were generated by first producing lentiviral vectors via cotransfection of HEK293T cells with pCW-Cas9 (gift from Eric Lander and David Sabatini; Addgene no. 50661) (27), packaging vector psPAX2 (gift from Didier Trono; Addgene no. 12260), and a plasmid encoding VSV G (pMDG; gift from James Cunningham, Brigham and Women’s Hospital, Boston) in a 4:3:1 ratio using the transfection reagent JetPRIME (Polyplus). Lentiviruses were harvested 48 h posttransfection, filtered with a 0.45-μm filter, and then used to infect HT1080 cells in the presence of 8 μg/ml Polybrene. Following puromycin selection, monoclonal cell populations were isolated via ring cloning and expanded. Several clones were induced in 1 μg/ml Dox over 4 days and were assessed for Flag-Cas9 expression by immunoblotting. Uninduced clones were also assessed for leaky expression of Flag-Cas9 by immunofluorescence and immunoblotting. One clone with strong expression of Flag-Cas9 upon Dox induction and no detectable expression in the absence of Dox was ultimately chosen for further experimentation.

Paired gRNA constructs were generated as described previously (28). Briefly, two gRNAs targeting the gene of interest were cloned into the lentivirus sgRNA(MS2)_zeo backbone (gift from Feng Zhang; Addgene no. 61427) (44) using pDonor sU6 (gift from Andrea Ventura; Addgene no. 69351) (28). The gRNA sequences were as follows: for NPC1, ACGCCTGTAATGCCACCAAC and CACAAGCAAAAACGCCATGT; for VPS11, GCGCTTCGTTTTCTTCGACA and GTGTGTCACCCGTAGTTTGT; for VPS16, GCAGAGTATATATCGAGCAC and GATGGTGCTGTACTGGTTTA; for VPS18, GTCCTCTACGTGAACCGAAA and GGACATGAACCGCTTCGATC; for VPS33A, GAACCTAAACGTGTTGCGCG and GAATACCTAACTGGACCCTT; for VPS3, GTGGTAGACGAAGCAGTCGT and CATGAGGAAGCGTTTGCACT; for VPS8, GTTGGAGGAGTATCAACTTG and GGAAGCGCTCATTGTACATA; for VPS39, TATAGATCCCACCCATGTGA and GTCAAGCACCTCACCGCTCA; and for UVRAG, ATCTTCGGAACATTGCTGCC and GATATCTGAGGGGCACTTGT.

Lentiviruses expressing dual gRNAs were produced by cotransfecting HEK293T cells with the lentiviral vector containing the dual gRNAs, psPAX2, and pMDG in a 4:3:1 ratio, as described above. Lentiviruses were then used to infect the monoclonal Cas9-inducible HT1080 cells in the presence of 8 μg/ml Polybrene, followed by zeocin selection. Cas9 expression was induced by seeding the CRISPR cell lines in 6-well plates to approximately 30% confluence in the presence of 1 μg/ml Dox. Untreated cells were kept as controls. Cells were allowed to proliferate for 96 h, at which point they were reseeded for further experimentation in the absence of Dox.

Genomic DNA extraction was performed by lysing cell pellets in 50 mM NaOH at 95°C for 20 min, followed by neutralization with 1 M Tris (pH 7.0) to a final concentration of 0.8 M (pH 8.3). Samples were centrifuged at 17,000 × *g* for 5 min, and supernatant was transferred to new tubes. Amplicons containing gRNA target regions were generated by PCR using Herculase II fusion DNA polymerase (Agilent). Surveyor endonuclease assays were performed by incubating PCR products in 1× NEBuffer2 with thermocycler conditions of 95°C (10 min), 95 to 85°C (ramp rate, −2°C/s), and 85 to 25°C (ramp rate, −0.3°C/s) to allow DNA duplexes to form. Due to the heterogeneity of the cell population from which DNA was extracted, both homoduplexes and heteroduplexes of DNA could form. Heteroduplexes, which indicate that targeting of the gene resulted in indels, were then digested with T7 endonuclease I (NEB) at 37°C for 1 h. Digested products were resolved by agarose gel electrophoresis and visualized under UV light.

### UVRAG cloning and generation of a polyclonal cell line expressing gRNA-insensitive UVRAG.

Synonymous mutations of PAM sequences on mCherry-UVRAG cDNA (gift from Do-Hyung Kim; Addgene no. 86743) (23) were performed using overlapping PCR and confirmed by Sanger sequencing. Further cloning was done using this PAM-mutated UVRAG construct. C2 and 269–442 deletions were generated using overlapping PCR and confirmed by Sanger sequencing and immunoblotting. All mCherry-UVRAG constructs were cloned into the pLV-EF1a-IRES-Blast (gift from Tobias Meyer; Addgene no. 85133) (45) lentiviral vector.

UVRAG rescue cell lines with add-back of WT or mutant UVRAG were generated by transducing UVRAG CRISPR cells with the mCherry-UVRAG constructs. Lentiviruses were generated as described above and used to infect UVRAG CRISPR cells in the presence of 8 μg/ml Polybrene, followed by selection in blasticidin. Expression of mCherry-UVRAG in the cell lines was validated by microscopy, flow cytometry, and immunoblotting.

### Virus-like-particle production and viral entry assays.

EBOV virus-like-particles (VLPs) were produced by cotransfecting HEK293T cells with plasmids encoding EBOV NP, EBOV VP40 fused to β-lactamase, or GFP (kind gifts from Lijun Rong, University of Illinois) and the viral envelope protein (mucin-deleted [Δmuc] EBOV GP, EBOV/Bundibugyo ebolavirus [BUDV]/Sudan ebolavirus [SUDV]/Taï Forest ebolavirus [TAFV]/Reston ebolavirus [RESTV] GP, or the glycoproteins of Marburg virus [MARV], VSV, or Junin virus [JUNV]) (all plasmids were kind gifts from James Cunningham, Brigham and Women’s Hospital) using JetPRIME transfection reagent at a 1:1:1.15 ratio. Virus-containing supernatants were collected and concentrated by ultracentrifugation (20,000 rpm, 1.5 h, 4°C; Beckman Coulter Optima XPN-100, SW32Ti rotor) through a 20% (wt/vol) sucrose cushion. Viruses were resuspended in phosphate-buffered saline (PBS) and stored at −80°C.

Entry assays were performed by seeding HT1080 cells in 48-well plates at approximately 90% confluence. VLPs were added, and plates were centrifuged at 200 × *g* for 30 min at 4°C and then incubated at 37°C for 3 h. Cells were washed with serum-free DMEM and loaded with CCF2-AM (Invitrogen) according to kit instructions for 1 h at room temperature. The staining solution was supplemented with 250 μM probenecid (Sigma). Following staining, cells were washed once in PBS and trypsinized, and cleavage of CCF2 was analyzed using a FACSCelesta flow cytometer (BD Biosciences). Stained cells were defined as cells that were positively shifted compared to unstained cells (emission in a 525/50 filter), and β-lactamase-positive (successful reporter release post-VLP fusion) cells were defined as cells that were positively shifted (emission in a 450/50 filter) compared to stained, uninfected cells.

### Immunofluorescence and microscopy.

HT1080 cells were seeded onto 18-mm coverslips and grown to approximately 60% confluence. Fusion-deficient (GP^F535R^) ΔMuc EBOV VLPs containing VP40-GFP were spinoculated onto cells (200 × *g*, 30 min, 4°C) to allow cell surface binding but not internalization. Cells were shifted to 37°C for 3 h to allow infection to proceed, followed by fixation in formalin, permeabilization with 0.5% Triton X-100, and blocking in 20% fetal bovine serum (FBS) for 30 min. Cells were then incubated in an NPC1 antibody (5-μg/ml concentration) for 1 h, followed by incubation with an Alexa Fluor 647-conjugated secondary antibody (Thermo) for 1 h, Hoechst staining (1 μg/ml; Thermo), and mounting in PermaFluor aqueous mounting medium (Thermo). Immunofluorescence images were captured on an LSM800 Zeiss confocal microscope using a 63×/1.4 oil plan Apochromat objective. Fifteen to twenty z-stacks were acquired per image.

### Immunoblots.

Cells were washed in PBS and then lysed in buffer containing 1% Triton X-100, 0.1% NP-40, 150 mM NaCl, and 10 mM Tris-HCl (pH 7.4) supplemented with a protease inhibitor cocktail (Cell Signaling). Protein concentration was determined using a bicinchoninic acid (BCA) assay following the manufacturer’s protocol (Thermo). Equal amounts of protein were resolved by sodium dodecyl sulfate-polyacrylamide gel electrophoresis (SDS-PAGE) and transferred to polyvinylidene difluoride (PVDF) membranes. Proteins were detected using appropriate primary antibodies (1 μg/ml concentration) and horseradish peroxidase (HRP)-conjugated secondary antibodies and visualized using chemiluminescence according to manufacturer protocol (Bio-Rad Clarity ECL substrate).

### Image and statistical analysis.

Image analysis was performed using Imaris software (Bitplane) as described previously (18). Briefly, VLPs were modeled as GFP^+^ puncta with a diameter of 0.3 μm or greater. For determination of colocalization with NPC1, VLPs were assigned colocalization values based on intensity correlation to the Alexa Fluor 647 channel (NPC1), and the colocalization threshold was manually determined for each experiment. The same threshold was used for both induced and noninduced cells in each experiment. The percentage of VLPs colocalized to NPC1 was then determined by dividing the number of VLPs above the colocalization threshold by the total VLP count per cell. For determination of the number of internalized VLPs per cell, cell boundaries were modeled using cell fluorescence background intensity (647 nm), and VLPs were modeled as described above.

Data are presented as means and standard deviations. Statistical analysis was performed by an unpaired *t* test with Welch’s correction, and statistical significance was determined using the Holm-Šídák method, unless otherwise indicated.
